# Whole Genome Duplication Events Likely Contributed to the Aquatic Adaptive Evolution of Parkerioideae

**DOI:** 10.3390/plants13040521

**Published:** 2024-02-14

**Authors:** Meng Wang, Rui Zhang, Jiang-Ping Shu, Xi-Long Zheng, Xin-Yi Wu, Jian-Bing Chen, Mei-Na Wang, Hui Shen, Yue-Hong Yan

**Affiliations:** 1 Shenzhen Key Laboratory for Orchid Conservation and Utilization, Key Laboratory of National Forestry and Grassland Administration for Orchid Conservation and Utilization, The National Orchid Conservation Center of China and the Orchid Conservation and Research Center of Shenzhen, Shenzhen 518114, China; wangmeng65@126.com (M.W.); jiangping_shu@163.com (J.-P.S.); xin-shenhaiyu@163.com (X.-Y.W.); cjb@cnocc.cn (J.-B.C.); wangmn@cnocc.cn (M.-N.W.); 2 Eastern China Conservation Centre for Wild Endangered Plant Resources, Shanghai Chenshan Botanical Garden, Shanghai 201602, China; piaoyao00520@163.com (R.Z.); shenhui@csnbgsh.cn (H.S.); 3 Shanghai Key Laboratory of Plant Functional Genomics and Resources, Shanghai Chenshan Botanical Garden, Shanghai 201602, China; 4 Guangdong Provincial Key Laboratory for Plant Epigenetics, College of Life Sciences and Oceanography, Shenzhen University, Shenzhen 518060, China; 5 School of Traditional Medicine Materials Resource, Guangdong Pharmaceutical University, Yunfu 527322, China; zhengxl2012@sina.com

**Keywords:** Parkerioideae, whole genome duplication, adaptive evolution, high salinity adaptation, transcriptome

## Abstract

As the only aquatic lineage of Pteridaceae, Parkerioideae is distinct from many xeric-adapted species of the family and consists of the freshwater *Ceratopteris* species and the only mangrove ferns from the genus *Acrostichum*. Previous studies have shown that whole genome duplication (WGD) has occurred in Parkerioideae at least once and may have played a role in their adaptive evolution; however, more in-depth research regarding this is still required. In this study, comparative and evolutionary transcriptomics analyses were carried out to identify WGDs and explore their roles in the environmental adaptation of Parkerioideae. Three putative WGD events were identified within Parkerioideae, two of which were specific to *Ceratopteris* and *Acrostichum*, respectively. The functional enrichment analysis indicated that the lineage-specific WGD events have played a role in the adaptation of Parkerioideae to the low oxygen concentrations of aquatic habitats, as well as different aquatic environments of *Ceratopteris* and *Acrostichum*, such as the adaptation of *Ceratopteris* to reduced light levels and the adaptation of *Acrostichum* to high salinity. Positive selection analysis further provided evidence that the putative WGD events may have facilitated the adaptation of Parkerioideae to changes in habitat. Moreover, the gene family analysis indicated that the plasma membrane H^+^-ATPase (*AHA*), vacuolar H^+^-ATPase (*VHA*), and suppressor of K^+^ transport growth defect 1 (*SKD1*) may have been involved in the high salinity adaptation of *Acrostichum*. Our study provides new insights into the evolution and adaptations of Parkerioideae in different aquatic environments.

## 1. Introduction

Ferns (monilophytes), with more than 10,000 extant living species globally, are a significant land plant lineage that occupies a wide diversity of land environments [1,2]. Some extant ferns have returned to the water, such as *Azolla* and *Salvinia* in Salviniaceae and Parkerioideae in Pteridaceae [3,4]. Pteridaceae comprises over 50 genera and more than 1000 species, about 400 species of which form the cheilanthoid clade and are characterized by their ability to thrive in xeric habitats [3]. On the contrary, Parkerioideae, one subfamily of Pteridaceae, has adapted to aquatic environments and is the only aquatic lineage of Pteridaceae [3]. More interestingly, Parkerioideae only includes two genera and about 12 species, but the two genera, *Ceratopteris* and *Acrostichum*, occupy freshwater habitats and marine intertidal zones, respectively. In addition, *Acrostichum* is the only mangrove genus among ferns [3]. The marine intertidal zones, where *Acrostichum* species have colonized, are constantly submerged in seawater, and one of the notable differences from the freshwater habitats of *Ceratopteris* is the high salinity [5]. It is of interest to understand how Parkerioideae diverged from Pteridaceae, which includes many xeric-adapted species, and returned to the water. Moreover, how *Acrostichum*, the only fern genus among mangroves, has adapted to marine intertidal zones characterized by high salinity is also of considerable interest.

Among the major vascular plant lineages, aquatic species evolved from terrestrial species by dealing with low light levels, reduced carbon and oxygen availability, and mechanical damage from waves [4]. For example, the formation of aerenchyma, the enhancement of glycolytic fluxes, and ethanolic fermentation have been interpreted as adaptations to low oxygen concentrations of aquatic habitats [3,4]. In addition, mangroves have had at least 27 independent origins from inland ancestors and have evolved a series of highly specialized characteristics to colonize marine intertidal zones, such as salt tolerance, aerial roots, and viviparous embryos [6,7,8]. Therefore, we can speculate that the aquatic Parkerioideae evolved from the terrestrial ancestors of Pteridaceae, and the mangrove *Acrostichum* originated from inland ancestors of Parkerioideae. Both evolutions involved specialized features to adapt to new environments, such as the aerenchyma of Parkerioideae and the salt tolerance of *Acrostichum* [3].

Whole genome duplication (WGD) is widespread and commonplace in plants. There have been 244 ancient WGDs inferred across Viridiplantae based on the vegetative transcriptomes of 1124 species [9]. Through extensive sampling, many lineages were found to have experienced numerous WGDs, such as Asterids [10], Fabaceae [11], Caryophyllales [12], and ferns [13]. WGDs provide plenty of genetic material for evolution through doubling the genome content and are recognized as an important evolutionary force for adaptation [14,15,16,17]. The fact that many WGDs have been clustered around some severe global environmental changes, such as the K-Pg boundary [18], is strong evidence for their roles. Additionally, gene families functioning in response to stress, which corresponds with the main environmental stress, were retained in multiple independent WGDs in angiosperms [15] and may have played a crucial role in adapting to stressful environments. 

The habitats of Parkerioideae, aquatic environments and unstable marine intertidal zones, are challenging environments, and WGDs may have occurred in this lineage and contributed to environmental adaptation. The evidences of mangroves and Alismatales, the largest clade of aquatic angiosperms, support the role of WGDs in adaptation to marine intertidal zones and aquatic habitats [4,6,19]. Among Parkerioideae, *Ceratopteris* species were believed to experience at least one WGD based on the genome of *Cer. richardii* and the transcriptomes of *Cer. thalictroides* [9,20,21]. This WGD was dated at 51~60 Mya with a Ks peak value of approximately 1.2~1.3 [20,21] and likely occurred after the divergence of *Acrostichum* and *Ceratopteris* based on their divergence time of 80.4 Mya [22] and the earliest *Acrostichum* fossil record of 66.0~72.1 Mya [23]. However, the relationship between this identified WGD and the divergence event needs more in-depth study. In addition, the mangrove *Aegiceras corniculatum* experienced a recent WGD, which complemented the formation of adaptive features for marine intertidal environments [19,24]. So, the mangrove genus *Acrostichum* may have also experienced WGDs, though this is yet to be confirmed. Up to now, researchers have yet to comprehensively explore WGDs within Parkerioideae, and it is still unclear whether WGDs were involved in the environmental adaptation of Parkerioideae.

Here, we identified WGDs and their roles within Parkerioideae using 13 transcriptomes, including four *Acrostichum* samples, eight *Ceratopteris* samples, and *Coniogramme japonica* as an outgroup. By conducting comparative and evolutionary transcriptomics analyses, such as phylogenetic analysis, WGD inference, positive selection detection, and gene family analysis, we aimed to (1) identify WGDs within Parkerioideae and place the identified WGDs on a phylogenetic tree, (2) explore the role of WGDs in environmental adaptation in Parkerioideae, and (3) analyze the salt-stress-response-associated gene families potentially involved in the high salinity adaptation of *Acrostichum*.

## 2. Results

### 2.1. Transcriptome Processing and BUSCO Assessment

Thirteen transcriptomes were collected, including four samples of *Acrostichum*, eight samples of *Ceratopteris*, and one sample of *Coniogramme japonica*, of which eleven were downloaded from the National Center for Biotechnology Information (NCBI) Sequence Read Archive (SRA) database and two were newly sequenced in this study (Table 1). The speciation of *Ceratopteris* species is complex [25,26,27,28,29], and some cryptic species are newly found in *Ceratopteris*, so we revised the samples of *Cer. pteridoides* (Wuhan, China; SRR4210088, SRR4210089) as *Cer. chingii* and the sample of *Cer. thalictroides* (Zhejiang, China; SRR2103737) as *Cer. gaudichaudii* var. *vulgaris* according to Yu et al. (2022) [29]. We obtained 127.74 Gb raw data in total, and assembled 46,939 (*A*. *aureum*, SRR2103733) to 319,024 transcripts (*Cer. richardii*, SRR13179619), got 39,037 (*A*. *aureum*, SRR2103733) to 258,884 unigenes (*Cer. richardii*, SRR13179619) after removing redundancy, and finally predicted 26,286 (*A*. *aureum*, SRR2103733) to 81,815 coding sequences (CDSs) (*Cer. richardii*, SRR13179619) (Supplementary Table S1). 

The assembly completeness of the transcriptomes was assessed by BUSCO (v.5.2.2) [30,31,32] with mapping to the core plant homologous genes of the viridiplantae_odb10 dataset [33]. Only 0.9~18.6% of the Benchmarking Universal Single-Copy Orthologs (BUSCOs) were missing, suggesting the acceptable coverage and high quality of these assemblies (Supplementary Table S1).

**Table 1:** Transcriptome data used in this study.

Species	Accession	Sampling Site	Ref
*Acrostichum aureum*	SRR1822234	Nansha, Guangzhou, China	[34]
	SRR2103733	Greenhouse of Shanghai Chen Shan Botanical Garden, Shanghai, China	[1]
	SRR6920722	Hainan, China	[35]
*Acrostichum speciosum*	SRR1822235	Qinglan Harbour, Wenchang, Hainan, China	[34]
*Ceratopteris thalictroides*	SRR1822236	Greenhouse of Sun Yat-sen University, Guangzhou, China	[34]
*Ceratopteris gaudichaudii* var. *vulgaris*	SRR2103737	Zhejiang, China	[1]
*Ceratopteris* sp.	SRR26877014	Nayang village, Hainan, China	This study
*Ceratopteris shingii*	SRR26877015	Jiangjun Mountain, Hainan, China	This study
*Ceratopteris chingii*	SRR4210088	Wuhan, China	[36]
	SRR4210089	Wuhan, China	[36]
*Ceratopteris richardii*	SRR10317164	Shanghai Institute of Plant Physiology and Ecology, Chinese Academy of Sciences, Shanghai, China	[37]
	SRR13179619	West Lafayette, IN, USA	[38]
*Coniogramme japonica*	SRR6920719	Zhejiang, China	[35]

### 2.2. Phylogenetic Analyses

Thirteen transcriptomes were used to infer orthogroups, and 94.5% of a total of 588,261 protein sequences were assigned to 48,099 orthogroups, 359 of which consisted entirely of single-copy genes. A maximum likelihood tree was reconstructed using the concatenated alignment of the 359 single-copy orthologous genes (OGs), clustering into two main clades as expected. The relationships among *Ceratopteris* species were similar to those found by Yu et al. (2022) [29]; *Cer. shingii* first split from other *Ceratopteris* species, and *Cer. chingii* and *Cer. gaudichaudii* var. *vulgaris* clustered into one subclade (Supplementary Figure S1). A low bootstrap value of 57 for the basal position of *Cer. shingii* was also observed, but high support values were shown in the previous phylogenetic trees based on multiple plastid regions [27,29]. Moreover, hybridization is common among *Ceratopteris* species, and the frequent hybridization may lead to the complex speciation of tetraploid *Ceratopteris* species [26,29]. *Cer. shingii* was reported as a tetraploid with 2n = 154 [27], and the complex speciation and evolution history of *Ceratopteris* species may be one reason for the low bootstrap value of its basal position, which needed additional study.

### 2.3. Inference of Whole Genome Duplication Events

**Figure 1:**
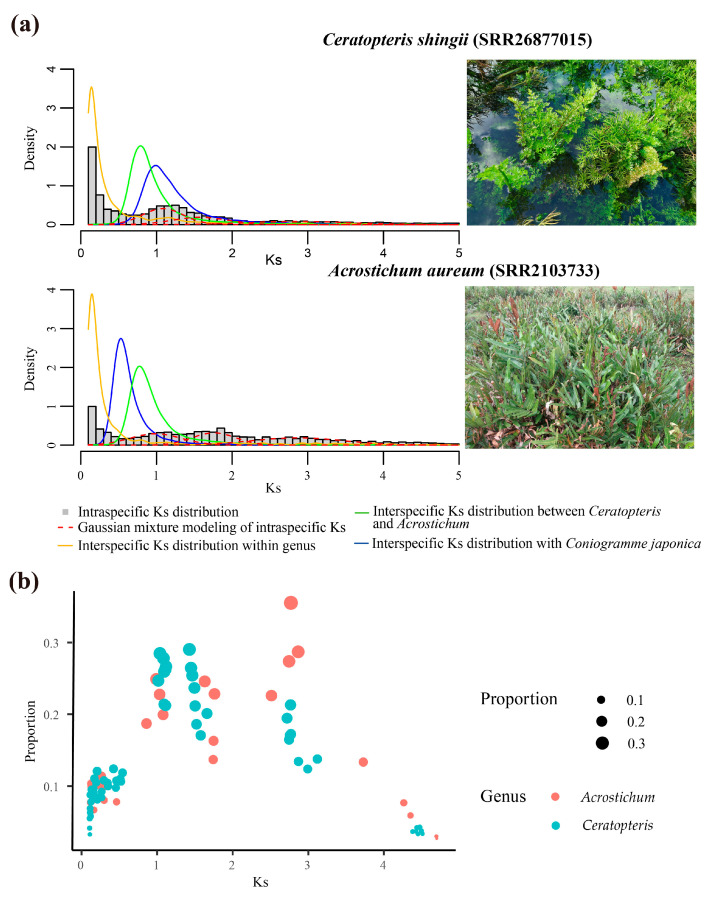
Intraspecific and interspecific Ks distribution and Gaussian mixture modeling result. (**a**) The integrated plots of the Ks distribution and Gaussian mixture modeling result. The intraspecific Ks distributions are represented by gray histograms. The interspecific orthologs’ Ks distributions within the genus (between each species within *Ceratopteris* and *Cer*. *shingii*, between each species within *Acrostichum* and *Acr*. *speciosum*), between genera (between each *Acrostichum* species and *Cer*. *shingii*, and each *Ceratopteris* species and *Acr*. *speciosum*), and with *Coniogramme japonica* (between each species and *Con*. *japonica*) are presented by yellow, green, and blue solid curves, respectively. The red dashed curves represent the Gaussian mixture modeling result. (**b**) The Gaussian mixture modeling result of intraspecific Ks distribution is summarized from Supplementary Table S2.

Intraspecific Ks analysis was used to infer whole genome duplication events (WGD events) by fitting the WGD Gaussian function with the R package mclust [39]. The putative WGD events of Ks values less than 2 were focused on in the following analysis, because higher Ks values are uncertain due to Ks saturation and stochastic effects [40]. Two putative WGD events of *Ceratopteris* with the mean Ks values of 1.43~1.66 and 1.02~1.12 and two putative WGD events of *Acrostichum* with the mean Ks values of 1.63~1.76 and 0.86~1.08 were detected (Figure 1 and Supplementary Table S2). The interspecific Ks distribution was applied to place putative WGD events in relation to lineage divergence. As expected, the interspecific Ks values within the genus were less than the Ks peaks of all putative WGD events, indicating that the species divergence within the genus likely occurred after WGD events. However, the interspecific Ks values between *Ceratopteris* and *Acrostichum*, *Ceratopteris* and *Con. japonica*, and *Acrostichum* and *Con. japonica* also had a similar result (Figure 1a and Supplementary Figure S2), suggesting that all putative WGD events preceded lineage divergence.

**Figure 2:**
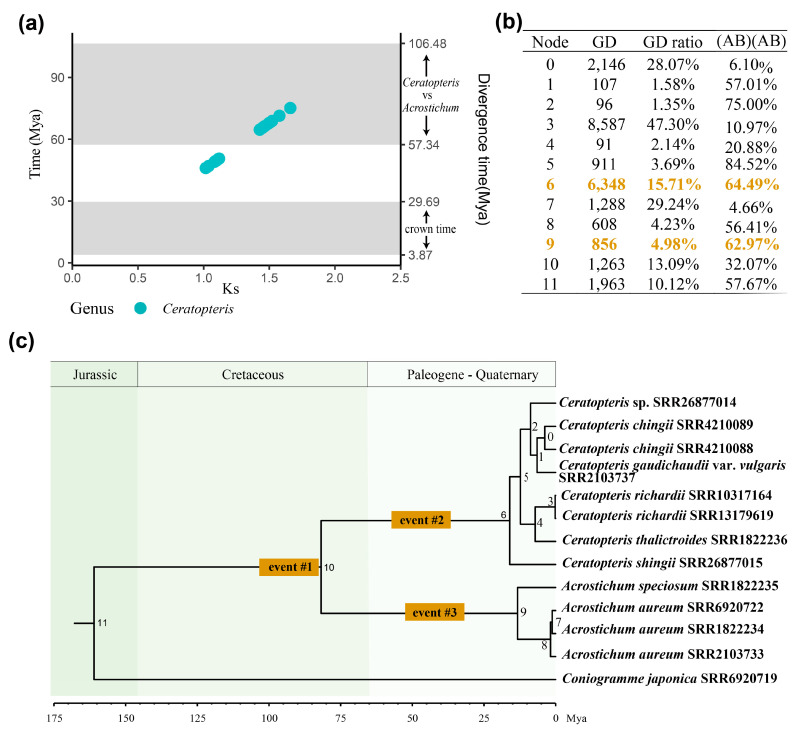
Three inferred WGD events and their position on the phylogenetic tree. (**a**) The time comparison between divergence and WGD events. The x-axis is the Ks value of putative WGD events inferred by Ks analysis. The colored points are the dates of putative WGD events, visualized from Supplementary Table S3. The gray bars are the estimated divergence time, corresponding to the divergence time between *Ceratopteris* and *Acrostichum* (57.34~106.48 Mya), and the crown time of *Ceratopteris* and *Acrostichum* (3.87~29.69 Mya), summarized from Supplementary Figure S3. (**b**) Two putative WGD events inferred by the phylogenetic method. The corresponding node numbers are labeled around the nodes in Figure (**c**). The table also contains the number of duplicated gene families (GD), the ratio of GD to gene families (GD ratio), and the proportion of (AB)(AB)-type GD of each node. Given that a gene duplication event has occurred at the MRCA of lineages A and B, the GD retained in both A and B was recognized as an (AB)(AB)-type GD. The nodes that may have experienced WGD events are in yellow. (**c**) The position of three putative WGD events on the phylogenetic tree. The putative WGD event #1 was mainly inferred based on the Ks analysis. The putative WGD event #3 was inferred by the phylogenetic method, and its date needed further analysis.

To further explore the relationship among the putative WGD events and lineage divergence, estimations of the divergence time and dating of the WGD events were performed (Supplementary Figure S3 and Supplementary Table S3). The divergence time estimation was performed by MCMCTree of PAML (v.4.9j) [41] with two constraints, which were set according to Lehtonen et al. (2017) [22]. The putative WGD events of *Ceratopteris* inferred by Ks analysis were dated using the synonymous substitution rate of *Cer. thalictroides*, 11.04 × 10^−9^ synonymous substitutions per site per year, according to the absolute dating of Zhang et al. (2019) [21]. The time comparison between the lineage divergence and WGD events is summarized in Figure 2a, and the results show that the date of the putative WGD event with Ks values of 1.43~1.66 coincided with the divergence time between *Ceratopteris* and *Acrostichum*, indicating that this putative WGD event occurred in the most recent common ancestor (MRCA) of *Ceratopteris* and *Acrostichum.* The date of the putative WGD event with Ks values of 1.02~1.12 was earlier than the crown time of *Ceratopteris* and *Acrostichum*, but later than the lineage divergence time (Figure 2a), indicating one *Ceratopteris*-specific WGD event, which rejected the Ks analysis suggestion that all putative WGD events in *Ceratopteris* preceded the lineage divergence events. 

The Ks plots of *Acrostichum* also indicated that the two putative WGD events of *Acrostichum* with the mean Ks values of 1.63~1.76 and 0.86~1.08 preceded lineage divergences between *Ceratopteris* and *Acrostichum*, and *Acrostichum* and *Con. japonica* (Figure 1a and Supplementary Figure S2). Considering that the synonymous substitution rate of *Cer. thalictroides* is higher than the average rate for plants (6.1 × 10^−9^ synonymous substitutions per site per year) [21,42], the synonymous substitution rate of *Cer. thalictroides* is inappropriate for application in dating the putative WGD event of *Acrostichum*. The method of evolutionary rate correction developed by Wang et al. (2015) was used to infer the corrected Ks (denoted by Ks’) of the two putative WGD events in *Acrostichum* species [43], and the Ks’ of *Acrostichum* was relatively comparable with the Ks values of *Cer. thalictroides*. The detailed procedures are described in the Methods section. After performing the correction, the two putative WGD events of *Acrostichum* showed Ks’ values of 2.47~3.05 and 4.68~5.02, respectively (Supplementary Table S4). Compared with the fact that the putative WGD event with a Ks value of 1.43 in *Cer. thalictroides* was estimated to occur in the MRCA of *Ceratopteris* and *Acrostichum* (Figure 2a), the two putative WGD events of *Acrostichum* inferred in the Ks plots were more ancient. 

In addition, a phylogenetic method was also performed to detect and place putative WGDs within Parkerioideae. Four putative WGD events were inferred by the phylogenetic software Tree2GD (v.1.0.39) [10,44] following the criteria of Zhao et al. (2021) [11], of which one event was shared by Parkerioideae and *Con. japonica*, one putative WGD event occurred at the MRCA of *Ceratopteris* and *Acrostichum*, one was a *Ceratopteris*-specific WGD event, and one was an *Acrostichum*-specific WGD event (Figure 2b, Supplementary Figures S4 and S5). The first and second putative WGD signals were ignored due to limited sampling. Thus, among the putative WGD events inferred by Ks analysis, the *Ceratopteris*-specific WGD event was also supported by the phylogenetic method.

Finally, three putative WGD events were inferred within Parkerioideae: one was shared by *Ceratopteris* and *Acrostichum* (event #1, with Ks value 1.43~1.66 in *Ceratopteris*), and the other two were specific to *Ceratopteris* (event #2, with Ks value 1.02~1.12) and *Acrostichum* (event #3, inferred by the phylogenetic method), respectively (Figure 2c). The WGD event #2 was supported by both Ks analysis and the tree-based method. The signal of WGD event #1 was also detected using the tree-based method, but was less reliable due to limited sampling. The WGD event #1 was mainly inferred by Ks analysis, so the subsequent analyses focused on the WGD event #2 and event #3.

### 2.4. Functional Enrichment Analysis 

**Figure 3:**
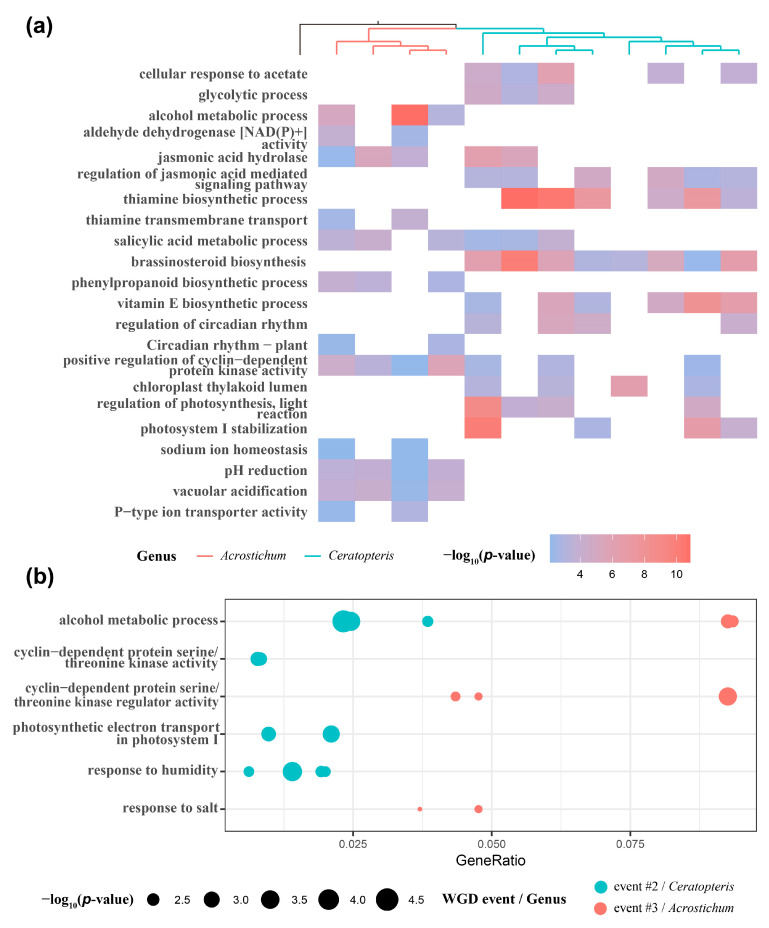
Functional enrichment result of duplicated genes (GDs) and positively selected GDs. (**a**) The associated function of aquatic adaptation enriched from GDs of WGD event #2 and event #3. (**b**) The enriched function from positively selected GDs contributed to the adaptation of *Ceratopteris* and *Acrostichum* to different aquatic habitats. One point represents one sample.

Functional enrichment analysis has been widely applied to elucidate the functional retention bias of duplicated genes after WGD events. The duplicated genes of putative WGD event #2 and event #3 in each sample inferred by Tree2GD [10,44] were delivered to the clusterProfiler package, which performed the functional enrichment analysis [45]. Shared and different functional terms between *Ceratopteris*-specific WGD event #2 and *Acrostichum*-specific event #3 were observed (Supplementary Figure S6 and Supplementary Table S5). In both WGD event #2 and event #3, the possible responses to the low oxygen concentrations of aquatic habitats were found, such as the “glycolytic process” and “cellular response to acetate” in WGD event #2, and the “alcohol metabolic process“ and “aldehyde dehydrogenase [NAD(P)+] activity“ in WGD event #3 (Figure 3a) [46]. In addition to the metabolic, transport, and regulation process of carbohydrates, proteins, and lipids, those of the stress-related compounds were also enriched, such as jasmonic acid, salicylic acid, and thiamine in both WGD event #2 and event #3, brassinosteroid and vitamin E in WGD event #2, and phenylpropanoid in WGD event #3 (Figure 3a) [47,48,49,50,51,52,53]. More interestingly, we found some functional terms associated with circadian rhythm and cell cycle in both WGD event #2 and event #3, such as “positive regulation of cyclin-dependent protein kinase activity” (Figure 3a).

Moreover, some different functional terms enriched by each WGD event may indicate differences in the habitats of *Ceratopteris* and *Acrostichum* species. The duplicated genes involved in photosynthesis tended to be retained in *Ceratopteris* species, resulting in the enriched functional terms of “chloroplast thylakoid lumen”, “photosystem I stabilization”, and “regulation of photosynthesis, light reaction” (Figure 3a). In addition, the responses to salt stress of *Acrostichum*, growing in the marine intertidal zone under high salt concentrations, were found in the lineage-specific WGD event #3, including “sodium ion homeostasis”, “vacuolar acidification”, “P-type ion transporter activity”, “pH reduction”, and “proton-transporting V-type ATPase complex” [54,55] (Figure 3a). 

### 2.5. Positive Selection Detection

To investigate the genetic adaptations to the freshwater environment of *Ceratopteris* and high salt marine intertidal zone of *Acrostichum*, positive selection was detected in duplicated gene pairs of each putative WGD event using the Ka/Ks method, 3.11~12.54% of which were found to be under positive selection with a Ka/Ks > 1 (Supplementary Table S6). The functional enrichment analysis of the positively selected GDs was performed, and some functional terms related to habitats were also found in WGD event #2 and event #3 (Figure 3b and Supplementary Table S7). Compared with terrestrial plants, ethanolic fermentation is enhanced in aquatic plants to adapt to the low oxygen levels of aquatic habitats [4]. The functional term “alcohol metabolic process” was shared by most *Ceratopteris* in WGD event #2 and *Acrostichum* in WGD event #3 (Figure 3b), which may indicate genetic adaptation to aquatic habitats. The duplicated genes related to cell cycle were also under positive selection; the associated functional terms were enriched, such as “cyclin-dependent protein serine/threonine kinase activity” and “positive regulation of cyclin-dependent protein kinase activity” in WGD event #2, and “cyclin-dependent protein kinase holoenzyme complex” and “cyclin-dependent protein serine/threonine kinase regulator activity” in WGD event #3 (Figure 3b and Supplementary Table S7). Moreover, the functional terms of photosynthesis and “response to humidity” were only shared by most *Ceratopteris* samples in WGD event #2, and “response to salt” was only shared by most *Acrostichum* samples in WGD event #3 (Figure 3b and Supplementary Table S7), similar to the functional enrichment analysis results of duplicated genes.

### 2.6. Gene Family Analysis

High salinity, mainly caused by high Na^+^ concentrations, is a significant challenge for the mangrove species *Acrostichum* living in the marine intertidal zone [24]. Na^+^ is generally not essential for plants, and a high Na^+^ level in the cytoplasm is toxic, causing K^+^ deficiency and inhibiting enzyme activity [56,57]. To prevent the accumulation of Na^+^ in the cytosol, sodium/hydrogen antiporters (*NHX*) are needed to transport Na^+^ from the cytosol to the vacuole or outside of the cell, driven by the H^+^ electrochemical membrane gradient, which is generated by proton pumps [57,58]. The involved proton pumps include plasma membrane H^+^-ATPase (*AHA)*, vacuolar H^+^-ATPase (*VHA),* and pyrophosphate-energized vacuolar membrane proton pump 1 (*AVP1*) [59,60,61]. Moreover, the key GO terms of “sodium ion homeostasis” and “vacuolar acidification” were enriched from the duplication of suppressor of K^+^ transport growth defect 1 (*SKD1*) and *VHA*, respectively. The expression of *ZmSKD1* in *Zea mays* was markedly up-regulated by salt stress, and its overexpression in tobacco plants enhanced their salt tolerance [62]. Thus, we chose these five gene families (*AHA*, *AVP1*, *NHX*, *SKD1*, *VHA*), which are involved in the response to salt stress, to explore the high salinity adaptation of *Acrostichum.*

**Figure 4:**
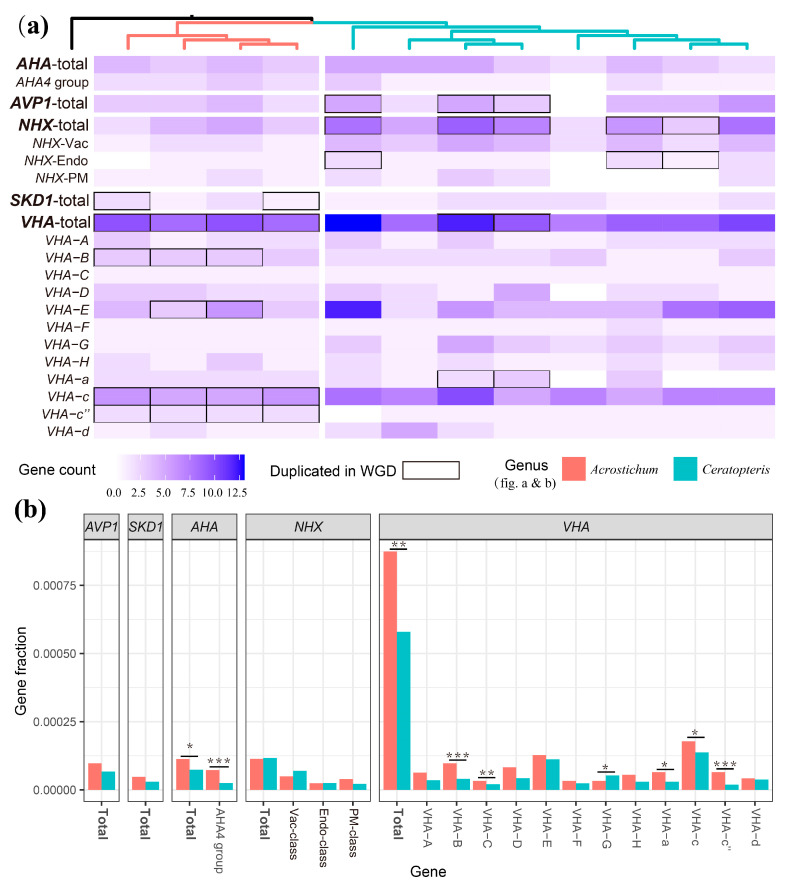
Gene copy counts, duplicates, and gene fraction of five target genes. (**a**) A heatmap presents the gene copy counts. The total number of *VHA* genes was much larger than the other genes, leading to discordant visualization, so a third of the total *VHA* number is presented in the heatmap. The black frames represent the duplicated genes. (**b**) The gene fraction of each gene in *Acrostichum* and *Ceratopteris*. *p*-values were calculated by independent two-sample *t*-test. * *p*-value < 0.05, ** *p*-value < 0.01, *** *p*-value < 0.001.

These five target gene families of all of the transcriptomes were identified through BLASTP (v.2.13.0) and HMMER (v.3.1b2) (http://hmmer.org/) (Supplementary Table S8). A total of 548 genes were identified as putative target genes, including 47 *AHA* genes, 46 *AVP1* genes, 64 *NHX* genes, 20 *SKD1* genes, and 371 *VHA* genes (Figure 4a and Supplementary Table S9). The subfamilies of *AHA*, *NHX*, and *VHA* were further classified based on the maximum likelihood phylogenetic trees constructed in this study (Supplementary Figures S7–S9) and previous research [58,63,64]. The *AHA* genes of Parkerioideae were divided into three clades, one of which was identified as the *AHA4* group, and the other two clades could not be identified due to sample limitations (Supplementary Figure S7). The *AHA4* group of Parkerioideae was further divided into two subclades, one of which included genes of both *Acrostichum* and *Ceratopteris*, while the other only consisted of *Acrostichum* (Supplementary Figure S7). The disrupted *AHA4* gene of an *Arabidopsis* mutant increased its sensitivity to salt stress [54], and expressing a constitutively activated H^+^-ATPase (ΔPMA4) in *Nicotiana tabacum* increased its salt tolerance [65], so the extra subclade of *Acrostichum* in the *AHA4* group may be related to high salinity adaptation. 

To investigate the role of WGD events in the high salinity adaptation of *Acrostichum*, the duplications of the five target gene families after WGD event #2 and event #3 were determined (Figure 4a and Supplementary Table S10). The duplicated genes of *AVP1*, Endo-class *NHX*, and *VHA-a* were retained in *Ceratopteris*, while *SKD1*, *VHA-B*, *VHA-E*, *VHA-c*, and *VHA-c*” were retained in *Acrostichum* (Figure 4a and Supplementary Table S10). The retaining of *SKD1* and *VHA* after *Acrostichum*-specific WGD event #3, which was estimated to occur before the species differentiation of *Acrostichum*, may have been involved in its high salinity adaptation.

The copy number and gene fraction of the five target genes were also considered (Figure 4 and Supplementary Table S9). The CDS number of *Acrostichum* (26,286~34,310, an average of 31,061) was significantly less than *Ceratopteris* (35,399~81,815, an average of 53,115) (*t*-test, *p*-value = 0.0077), so the gene fraction of each gene was calculated for the comparison between *Acrostichum* and *Ceratopteris*. For each target gene, the gene fraction for a species was calculated by the number of target genes divided by the number of total genes in a species, and the average gene fraction of the species in each genus was the gene fraction of each genus. The results showed that the gene fractions of *AHA*, *AHA4* group, *VHA*, *VHA-B*, *VHA-C*, *VHA-a*, *VHA-c*, and *VHA-c’’* in *Acrostichum* were significantly larger than that in *Ceratopteris*, while the gene fractions of *VHA-G* in *Acrostichum* were significantly lower than those in *Ceratopteris* (*t*-test, *p*-value < 0.05) (Figure 4b). The gene fractions of the other genes showed no significant difference between *Acrostichum* and *Ceratopteris*. Thus, the greater gene fractions of *AHA* and *VHA* may have contributed to the high salinity adaptation of *Acrostichum*.

## 3. Discussion

### 3.1. Three Putative WGD Events Occurred within Parkerioideae

Parkerioideae is the only aquatic lineage of Pteridaceae and comprises the only mangrove genus among ferns [3]. Therefore, Parkerioideae is useful to investigate how ferns adapt to aquatic environments and marine intertidal zones. Among angiosperms, WGDs were identified in Alismatales and mangroves, and were considered to contribute to the adaptation to aquatic habitats and marine intertidal zones [4,6,19]. However, only a few studies have been carried out concerning the WGDs of Parkerioideae, and only one WGD was identified within Parkerioideae, which likely occurred in *Ceratopteris* based on the evidence of the genome of *Cer. richardii* and the transcriptomes of *Cer. thalictroides* [9,20,21]. In this study, we used 13 transcriptomes, covering all genera of Parkerioideae, and two methods (Ks analysis and the phylogenetic method) to infer the WGD events of Parkerioideae.

Three putative WGD events were inferred within Parkerioideae (Figure 2c). One was shared by *Ceratopteris* and *Acrostichum* (event #1), and the other two were specific to *Ceratopteris* (event #2) and *Acrostichum* (event #3), respectively. The Ks peak around 2.51~3.12 was also observed in the Ks plots of *Ceratopteris* (Figure 1, Supplementary Figure S2, Supplementary Table S2), which was close to the ancient WGD in the ancestry of Polypodiales (PTERα, with a Ks value of 3.07 in *Cer. thalictroides*) [9]. The phylogenetic method also showed a signal of large-scale gene duplication at the root node (Figure 2a and Supplementary Figure S4), which was shared by Parkerioideae and *Con. japonica*, but we were unable to identify whether it was the WGD event PTERα due to limited sampling. In addition, we primarily focused on the WGD events within Parkerioideae, so the putative WGD event with a Ks value of 2.51~3.12 and/or at the root node, which was beyond Parkerioideae, was ignored in this study. Moreover, the limited sampling, only including one sample of the outgroup, also made the signal of WGD event #1 in the phylogenetic method lack reliability, especially in the gene families where the outgroup was absent. So, the WGD event #1 was mainly inferred by Ks analysis and needs more research. The putative WGD event #3 also needs more data, because it was inferred only by the phylogenetic method and might be affected by the lack of basal *Acrostichum* species.

Among the three putative WGD events within Parkerioideae, the Ks value of WGD event #2 was close to the previously inferred WGD (CERAα), the Ks value of which was around 1.08~1.3 [9,20]. The placement of WGD event #2 on the phylogenetic tree was also in accordance with the previous result of the genome of *Cer. richardii* [20]. Putative WGD event #1 and event #3 were newly inferred in this study, which confirmed our hypothesis that more than one WGD occurred within Parkerioideae.

### 3.2. The Synonymous Substitution Rate Varied between Ceratopteris and Acrostichum 

When the WGD events were inferred, we also observed a contradiction between the Ks analysis and the placement of WGD events on the phylogenetic tree. The Ks peaks of WGD events in *Ceratopteris* were greater than the Ks peak of the lineage divergence event between Parkerioideae and *Con. japonica* (Figure 1 and Supplementary Figure S2), generally indicating that the inferred WGD events of *Ceratopteris* occurred before the divergence of Parkerioideae and *Con. japonica* [9]. However, the phylogenetic-tree-based method, the dating results of the WGD events and divergence time, contradicted the suggestion of the above Ks analysis that all putative WGD events of *Ceratopteris* preceded lineage divergence events (Figure 2). We first eliminated methodological errors because the Ks peaks of WGD events in *Ceratopteris* and the divergence of *Ceratopteris* and *Acrostichum* were consistent with previous studies [20,21,34]. Then, the heterogeneous evolutionary rates among Pteridaceae were considered [66]. *Cer. thalictroides* had an accelerated evolutionary rate, which was higher than the average rate for plants [21]. The rapid evolutionary rate of *Cer. thalictroides* could be expected to produce a greater Ks value of the WGD event. For example, the Ks value of the WGD event PTERα in *Cer. thalictroides* was 3.07, which was greater than that of some other species in the same family, e.g., *Cryptogramma acrostichoides* (Ks value of 1.62), *Pteris vittata* (Ks value of 2.09), and *Pityrogramma trifoliata* (Ks value of 2.14) [9]. Therefore, the greater Ks values of WGD events in *Ceratopteris* species than lineage divergences could be explained by their accelerated evolutionary rate.

The Ks peaks of WGD events in *Acrostichum* were also greater than the Ks peaks of the lineage divergence events between Parkerioideae and *Con. japonica*, but the indication that the two putative WGD events of *Acrostichum* occurred before the divergence of Parkerioideae and *Con. japonica* might be right. Due to the lack of synonymous substitution rate in *Acrostichum*, we performed the Ks correction of duplicated genes within *Acrostichum* species, enabling a rough comparison of intraspecific Ks values between *Ceratopteris* and *Acrostichum*. The corrected Ks values (denoted by Ks’) of the two putative WGD events in *Acrostichum* were around 2.47~3.05 and 4.68~5.02, respectively, and the one with the Ks’ values around 2.47~3.05 was close to the Ks value of PTERα in *Cer. thalictroides* [9]. The greater Ks’ values supported the results that the two inferred WGD events in *Acrostichum* were shared by Parkerioideae and *Con. japonica*, and also implied that the synonymous substitution rate of *Acrostichum* is much slower than that of *Ceratopteris*. However, the synonymous substitution rate of *Acrostichum* still requires more precise analysis. 

### 3.3. WGD Facilitated the Adaptation of Parkerioideae

Polyploids, a consequence of WGD, are removed quickly from the population due to detrimental effects, such as genomic instability, abnormalities in mitosis, and meiosis [67]. However, polyploids may have a better chance of surviving in harsh or extreme environments, and play key roles in adaptation to new environments, especially stressful environments [15,42,67,68] such as aquatic environments and unstable marine intertidal zones. 

Alismatales, the largest clade of aquatic angiosperms, experienced 18 putative WGDs, and at least 13 WGDs occurred among the aquatic lineages, supporting the role of WGDs in adaptation to aquatic habitats [4]. Compared with terrestrial environments, the aquatic habitats were characterized by low oxygen concentrations and reduced light conditions [4]. The enhancements of glycolytic flux and ethanolic fermentations have been found when plants respond to low oxygen [46]. In this study, the related functional terms of glycolytic process and ethanolic fermentations were enriched in WGD event #2 and event #3 (Figure 3a). In addition to the possible responses to the low oxygen concentrations of aquatic habitats, the enrichments of photosynthesis were also observed in WGD event #2 (Figure 3a), which may be involved in the adaptation to low light levels in aquatic habitats. Moreover, the duplicated genes related to the alcohol metabolic process were under positive selection in both WGD event #2 and event #3, as well as that of photosynthesis in WGD event #2. So, the WGD may have played a vital role in the adaptation of Parkerioideae to the low oxygen and light levels of aquatic habitats. 

Among mangroves, *Aegiceras corniculatum* experienced a WGD about 35 million years ago (Mya), and the gene families related to salt homeostasis, 14-3-3 and H^+^-ATPase protein-coding genes, were preferentially retained after this WGD [19]. The retained duplicated genes adapted mangroves to the wide salinity range of the marine intertidal zones [6]. In this study, the newly found WGD event #3 occurred before the key event of *Acrostichum* invading marine intertidal zones, and may have contributed to the adaptation to new habitats of *Acrostichum*. The enriched functional term “sodium ion homeostasis” and the correlated terms were only shared by most *Acrostichum* in WGD event #3 (Figure 3a). In addition, some duplicated genes related to the response to humidity were under positive selection in the *Ceratopteris*-specific WGD event #2, while those in response to salt were under positive selection in the *Acrostichum*-specific WGD event #3 (Figure 3b). These results revealed the contribution of WGD events to adaptation to aquatic habitats and marine intertidal zones in Parkerioideae.

### 3.4. High Salinity Adaptation of Acrostichum

Soil salinization can be caused by either certain natural factors or the combination of anthropogenic factors and fragile environments, and is becoming a serious and growing global problem [69,70]. Increasing soil salinity impairs the growth and development of plants. Halophytes can survive to reproduce in high-salinity environments with salt concentrations of around 200 mM NaCl or more. There are 587 halophytes in China, including three species of ferns, two of which are *Acrostichum* species [71]. In addition, since the sister clade *Ceratopteris* occupies freshwater habitats, *Acrostichum* species may have evolved a suite of traits and molecular mechanisms to thrive under high-salinity conditions [72,73,74]. Thus, the *Acrostichum* genus is an excellent sample for understanding high salinity adaptation in ferns. 

In this study, we found that the lineage-specific WGD event #3 played a crucial role in the high salinity adaptation of *Acrostichum* through maintaining sodium ion homeostasis. The GO term “sodium ion homeostasis” and related terms were enriched among duplicated genes, while the GO term “response to salt” was enriched among duplicated gene pairs under positive selection (Figure 3). We carried out an analysis of five gene families involved in the response to salt stress, and the results showed that the gene families *AHA*, *VHA*, and *SKD1* may have contributed to the high salinity adaptation of *Acrostichum*. The gene families *AHA* and *VHA* encode plasma membrane and vacuolar H^+^-ATPase, respectively, which provide energy for Na^+^ transport from the cytosol to vacuole or outside of the cell [57,58,61,75], playing a crucial role in plant salt tolerance [76,77,78,79]. Four duplicates of *AHA* genes after the recent WGD event were retained in the mangrove *Aegiceras corniculatum* [19]. However, the retained duplicates of the *AHA* gene were not detected in the recent WGD of *Acrostichum*, but the gene fraction of *AHA* in *Acrostichum* was significantly greater than that in *Ceratopteris* (Figure 4). The duplicated genes of *VHA* were retained in the recent WGD of *Acrostichum*, and the gene fraction of *VHA* in *Acrostichum* was significantly greater than that in *Ceratopteris* (Figure 4), which may have enhanced Na^+^ transport from the cytosol to the vacuole, coinciding with the high salt composition of the vacuolars in leaves [72]. Moreover, the *SKD1* gene showed up-regulated expression under salt stress in *Zea mays* and enhanced the salt tolerance of tobacco plants when overexpressed [62]. In this study, the duplicated genes of *SKD1* were retained in the recent WGD of *Acrostichum*. However, transcriptomes may lose some copies of the gene families, so determining the molecular mechanism of high salinity adaptation in *Acrostichum* requires more genomic data and comparative analyses.

## 4. Materials and Methods

### 4.1. Transcriptome Data Collection, Processing, and BUSCO Assessment

Thirteen transcriptome samples from nine species were obtained, including four mangrove samples of *Acrostichum*, eight freshwater samples from *Ceratopteris*, and one terrestrial *Coniogramme japonica* sample as the outgroup. Of the 13 transcriptomes, 11 were RNA-seq raw reads downloaded from the National Center for Biotechnology Information (NCBI) database [1,34,35,36,37,38], and the remaining 2, *Cer. shingii* and *Ceratopteris* sp., were newly sequenced in this study (Supplementary Table S1). For transcriptome sequencing, the leaves of *Cer. shingii* and *Ceratopteris* sp. were collected separately from Jiangjun Mountain and Nayang village of Hainan province, China. RNA isolation, library preparation and sequencing were performed on the Illumina HiSeq platform by Novogene (Shanghai, China).

The RNA-seq raw reads from NCBI were extracted in FASTQ format files using the SRA Toolkit (v.2.10.8) [80], and then the FASTQ files, together with the two newly sequenced samples, were sent to Trimmomatic (v.0.39) [81] to remove adapters and low-quality regions. The clean data were used for de novo assembly by Trinity (v.2.11.0) [82] with min_kmer_cov set as 2. The assembled transcripts were delivered to cd-hit (v.4.8.1) [83] to remove redundancy with a sequence identity threshold of 95%, and the obtained non-redundant unigenes were used to predict coding sequences (CDSs) by TransDecoder (v.5.5.0) (https://github.com/TransDecoder/ (accessed on 11 November 2020)). The assembly completeness was evaluated by BUSCO (v.5.2.2) [30,31,32] with the core plant homologous gene database [33] (viridiplantae_odb10 dataset, accessed on 14 March 2023).

### 4.2. Orthology Inference and Phylogenetic Analyses

The protein sequences of all 13 transcriptomes were used to infer orthogroups through OrthoFinder (v.2.5.2) [84] with default settings, resulting in 359 single-copy orthologous genes (OGs). Each single-copy ortholog was aligned by MUSCLE v3.8.31 [85], and conserved blocks were selected using Gblocks (v.0.91b) [86,87]. Then, a supermatrix was merged from selected blocks of all of the single-copy OGs and used to reconstruct a phylogenetic tree. The maximum likelihood tree was constructed by RaxML (v.8.2.12) [88] under the best-fit model PROTGAMMAIJTTF found by ProtTest (v.3.4.2) [89], with *Coniogramme japonica* as an outgroup and 1000 bootstrapping repeats. 

### 4.3. Inference of Whole Genome Duplication

Ks analysis and the tree-based method were applied to infer potential WGD events. For the Ks analysis, the synonymous substitution distributions of intraspecific paralog pairs and interspecific ortholog pairs were both estimated. The former plots were used to infer putative WGD events, and the latter plots were used to place putative WGD events in relation to lineage divergence. To calculate the Ks distribution of intraspecific paralog pairs, each transcriptome’s protein sequences were used to perform all-against-all BLASTP (v.2.9.0) with an E-value cutoff of 1e-10^−10^. Then, an efficient script KSPlotter.py (https://github.com/EndymionCooper/KSPlotting (accessed on 10 December 2020)) was employed to build gene families, filter paralog pairs, align each paralog pair by MUSCLE (v.3.8.31) [85], calculate the Ks value with codeml of PAML (v.4.8a) [41], and reduce redundant Ks values. The Ks distribution of interspecific ortholog pairs was performed with similar procedures by wgd [90]. Only the Ks values between 0.1 and 5 were retained for the following analysis to avoid incorporating recent duplications and old substitution saturation. The peak in intraspecific Ks plots most likely corresponded to a putative WGD event and contained the WGD Gaussian function [91]. Therefore, the Gaussian mixture model was used in the R package mclust [39] to fit and confirm the WGD signature peaks. The Bayesian Information Criterion was used to select the maximum number of components to avoid overfitting.

For the tree-based method, Tree2GD (v.1.0.39) [10,44] was applied to identify putative WGD events by automatically performing multiple procedures with protein-coding sequences and a species tree as input files. The main steps were as follows. An all-against-all BLASTP of each pair species was first performed by DIAMOND (v.0.9.29.130) [92], and gene families were identified by clustering with PhyloMCL (v.2.0) [93]. Then, MUSCLE (v.3.8.31) [85] was used to perform multiple sequence alignments for each gene family, and the evolution of the gene family size was estimated by Dollo Parsimony. A gene tree of each gene family was constructed by IQ-TREE [94] with the JTT+G4 model and 1000 times ultrafast bootstrap. Finally, the gene trees were mapped with the species tree to discover gene duplication (GD) events. The GD number, the ratio of the GD number to the number of gene families (GD ratio), and the percent of GDs with the (AB)(AB) type (the GD shared by all branches) were considered to identify putative WGD events. The standards for identifying putative WGD events followed the findings of previous research [11], with a putative WGD event considered as such when it met either one of the following two criteria: (1) GD number > 450, GD ratio > 4.5%, and percent of (AB)(AB) type > 50%; or (2) GD number > 1000, GD ratio > 5%, and percent of (AB)(AB) type > 20%. The duplicated genes of each putative WGD event were extracted according to the Tree2GD result and used for the following functional enrichment and positive selection detection.

### 4.4. WGD Dating and Divergence Time Estimation

Based on the assumption that the number of synonymous substitutions increased approximately linearly with time, we were able to estimate the date of a putative WGD event with the formula divergence date = Ks/(2 × r). Absolute dating of one WGD event in *Cer. thalictroides* was performed by Zhang et al. (2019) and obtained the synonymous substitution rate r value of 11.04 × 10^−9^ synonymous substitutions per site per year [21]. Here, the r value of 11.04 × 10^−9^ synonymous substitutions per site per year and the formula divergence date = Ks/(2 × r) were used to infer the date of putative WGD events in *Ceratopteris*. 

The divergence time of each node was also estimated to clarify the relationship between the putative WGD events and species divergence. The MCMCTree of PAML (v.4.9j) [41] was applied to estimate the divergence time of each node, with the time constraint between *Coniogramme japonica* and the ancestor of *Acrostichum* and *Ceratopteris* set at 136.24~186.88 Mya and *Acrostichum* and *Ceratopteris* set at 56.56~106.23 Mya, according to Lehtonen et al. (2017) [22].

### 4.5. Synonymous Substitution Correction 

To place the putative WGD events of *Acrostichum*, which were inferred by Ks plots, we performed the Ks correction according to Wang et al. (2015) [43]. The corrected Ks values (denoted by Ks’) of the putative WGD events in *Acrostichum* were relatively comparable with the Ks values of *Cer. thalictroides*. The Ks’ values were obtained through three steps and additional CDS sequences of *Pteris vittate* downloaded from GigaScience Database [1]. Firstly, the Ks value of PTERα in *Pte. vittata* (Ks_(Pt)_ = 2.0878) was aligned to the peak location in *Cer. thalictroides* (Ks_(Ce)_ = 3.0713) [9], and then a correction coefficient *C* of duplicated genes in *Pte. vittata* was defined as *C*_Pt_ = Ks_(Ce)_/Ks_(Pt)_ = 1.471. Secondly, The Ks distributions of interspecific ortholog pairs between *Acrostichum* species and *Pte. vittata* and between *Cer. thalictroides* and *Pte. vittata* were performed by wgd [90]. The correction coefficient between the interspecific orthologs was defined as the algebra means of the correction coefficients between the duplicated genes in each species. For example, the correction coefficient of orthologs between *Pte. vittata* and *Cer. thalictroides* was defined as *C*_b(Pt, Ce)_ = (*C*_Pt_ + *C*_Ce_)/2; the Ks’ of the peak in the interspecific Ks plot was defined as Ks’_(Pt, Ce)_ = *C*_b(Pt, Ce)_ × Ks_(Pt, Ce)_. Finally, we aligned the peak Ks’ value of the interspecific distribution between *Pte. vittata* and *Acrostichum* species to that between *Pte. vittata* and *Cer. thalictroides*: Ks’_(Pt, Ce)_ = *C*_b(Pt, Ce)_ × Ks_(Pt, Ce) =_ Ks’_(Pt, Ac)_ = *C*_b(Pt, Ac)_ × Ks_(Pt, Ac)_. The correction coefficients of duplicated genes in *Acrostichum* species were defined as *C*_(Ac)_ = 2*C*_b(Pt, Ac)_ − *C*_Pt_ = 2 × ((*C*_b(Pt, Ce)_ × Ks_(Pt, Ce)_)/ Ks_(Pt, Ac)_) − *C*_Pt_, and the Ks’ values of duplicated genes within each *Acrostichum* species were obtained: Ks’_(Ac)_ = *C*_(Ac)_ × Ks_(Ac)_.

### 4.6. Inference of Positively Selected Genes

The ratio of nonsynonymous (Ka) to synonymous (Ks) substitution rates (Ka/Ks) usually indicates positive selection when Ka exceeds Ks (Ka/Ks > 1). The Ka/Ks ratios of duplicated gene pairs from putative WGD events were calculated by KaKs_Calculator (v.2.0) [95] with a parallel tool ParaAT (v.2.0) [96], and the main procedures were as follows. The protein sequences of each duplicated gene pair were first aligned by MUSCLE (v.3.8.31) [85] and then back-translated into the corresponding codon alignments using PAL2NAL [97]. The resulting codon alignments were formatted into an AXT format by ParaAT (v.2.0) [96] and used to calculate the Ka and Ks values by KaKs_Calculator (v.2.0) with default settings. The duplicated gene pairs were considered as being under positive selection if their Ka/Ks ratio was above 1, and were delivered to the subsequent functional enrichment.

### 4.7. Functional Enrichment Analysis

The protein sequences of each transcriptome were functionally annotated by eggNOG-mapper (v.2.0.6) [98] based on the eukaryotic dataset of eggNOG databases (v.5.0.1) [99] with default settings. The duplicated genes of each putative WGD event in each species were detected by Tree2GD (v.1.0.39) [10,44], and the ones under positive selection were used for functional enrichment by the clusterProfiler package [45] on the R (v.4.1.1) platform. The visualization was performed by ggplot2 (https://github.com/tidyverse/ggplot2 (accessed on 25 January 2021)), ggtree [100,101] (https://github.com/YuLab-SMU/ggtree (accessed on 16 April 2023)), aplot (https://github.com/YuLab-SMU/aplot (accessed on 16 April 2023)), and pheatmap (https://github.com/raivokolde/pheatmap (accessed on 25 January 2021)).

### 4.8. Gene Family Analysis

To further investigate the adaption of *Acrostichum* to the high salt conditions in marine intertidal zones, the associated duplicated genes of key GO terms (sodium ion homeostasis and vacuolar acidification) and the genes implicated in salt tolerance (plasma membrane and vacuolar proton pumps, sodium/hydrogen antiporter) were both analyzed. The associated duplicated genes, which were enriched for sodium ion homeostasis and vacuolar acidification, were identified as *SKD1* and *VHA* through a sequence similarity search at https://blast.ncbi.nlm.nih.gov/Blast.cgi (accessed on 19 April 2023). Moreover, the genes encoding sodium/hydrogen antiporter (*NHX*) and proton pumps (plasma membrane H^+^-ATPase, *AHA,* and pyrophosphate-energized vacuolar membrane proton pump 1, *AVP1*), which were found to play an essential role in salt tolerance [60,61], were also identified. 

The identification of target genes (*AHA*, *AVP1*, *NHX*, *SKD1*, *VHA*) was performed through BLASTP (v.2.13.0) and HMMER (v.3.1b2) (http://hmmer.org/) (Supplementary Table S10). The target genes of *Arabidopsis thaliana* were retrieved from the TAIR database [102] and used as a query to scan the protein sequences of 13 transcriptomes by BLASTP (v.2.13.0) with an e-value of 1e-5. The hmmsearch program in HMMER (v.3.1b2) was applied to search the protein sequences of 13 transcriptomes using the hidden Markov model (HMM) profiles, which were downloaded from the Pfam database [103], and the hits with a maximum e-value of 1e-5 were retained. The shared sequence of the BLASTP results and hmmsearch hits were submitted to Pfam web, checking the corresponding protein characteristic domains. The confirmed protein sequences were aligned by MAFFT (v.7.471). The alignment was delivered to IQ-TREE (v.2.0.3) [94] to construct a phylogenetic tree using the maximum likelihood method with the automatically determined substitution model (-m MFP) and 1000 bootstrapping repeats (VHA: -bb 1000; others: -b 1000).

## 5. Conclusions

To explore the history of WGD within Parkerioideae and its role in the adaptive evolution of Parkerioideae, we used Ks analysis, the phylogenetic method, and time comparison to identify WGD and its phylogenetic placement, and functional enrichment, positive selection, and gene family analysis to detect its contribution to the adaptation to the global environment and aquatic habitats. We identified three putative WGD events within Parkerioideae; one was shared by *Ceratopteris* and *Acrostichum* (event #1), and the other two were specific to *Ceratopteris* (event #2) and *Acrostichum* (event #3), respectively. WGD event #1 and event #3 were newly identified in this study. The WGD event #2 and event #3 played a critical role in the adaptation to different aquatic habitats of Parkerioideae, and the role of WGD event #1 needed more samples to obtain more reliable results. For example, the duplicated genes with a role in responding to low oxygen concentrations were retained in both WGD event #2 and event #3, and the duplicated genes involved in photosynthesis and sodium ion homeostasis were retained after WGD event #2 and event #3, respectively. Similar results were found in duplicated genes under positive selection. Furthermore, we found that *AHA*, *VHA* and *SKD1* may involve the high salinity adaptation of *Acrostichum*.

## Data Availability

The raw sequence data reported in this paper have been deposited in the SRA under Bioproject number PRJNA1029796. A supplementary dataset is available on figshare (10.6084/m9.figshare.25199498).

## Supplementary Materials

The following supporting information can be downloaded at: https://www.mdpi.com/article/10.3390/plants13040521/s1, Figure S1: Phylogenetic tree constructed with 359 single-copy orthologous genes; Figure S2: The plots of Ks analysis, including intraspecific and interspecific Ks analysis; Figure S3: Divergence time estimation using 359 single-copy OGs; Figure S4: Two WGD events inferred by the phylogenetic method; Figure S5: Phylogenetic gene trees of cluster 1109 (a) and cluster 1004 (b) constructed in Tree2GD; Figure S6: Functional enrichment heatmap of duplicated genes retained from two putative WGD events; Figure S7: Phylogenetic tree of *AHA* genes; Figure S8: Phylogenetic tree of *NHX* genes; Figure S9: Phylogenetic tree of *VHA* genes; Table S1: Summary of raw reads, assembly, and BUSCO assessment of transcriptome data; Table S2: Gaussian mixture modeling result of intraspecific Ks distribution; Table S3: Dating the putative WGD events with the synonymous substitution rate of *Ceratopteris thalictroides* according to the absolute dating of Zhang et al. (2019) [21]; Table S4: Correction of synonymous substitution in *Acrostichum*; Table S5: Functional enrichment result of genes from duplicated gene families inferred by Tree2GD; Table S6: Summary of positive selection detection in duplicated gene pairs by Ka/Ks ratio; Table S7: Functional enrichment result of duplicated genes under positive selection detected by Ka/Ks ratio; Table S8: Target genes and their identification; Table S9: Number of target gene copies in each species; Table S10: Duplicated target genes in each species.
