# Supplementary material for: Whole Genome Duplication Events Likely Contributed to the Aquatic Adaptive Evolution of Parkerioideae
Source: Plants (Basel). 2024 Feb 14;13(4):521. doi: 10.3390/plants13040521 (PMC10893450; doi:10.3390/plants13040521)
Supplement: Supplementary file 1 [file plants-13-00521-s001.zip › Supplementary_Figure20240214.pdf]

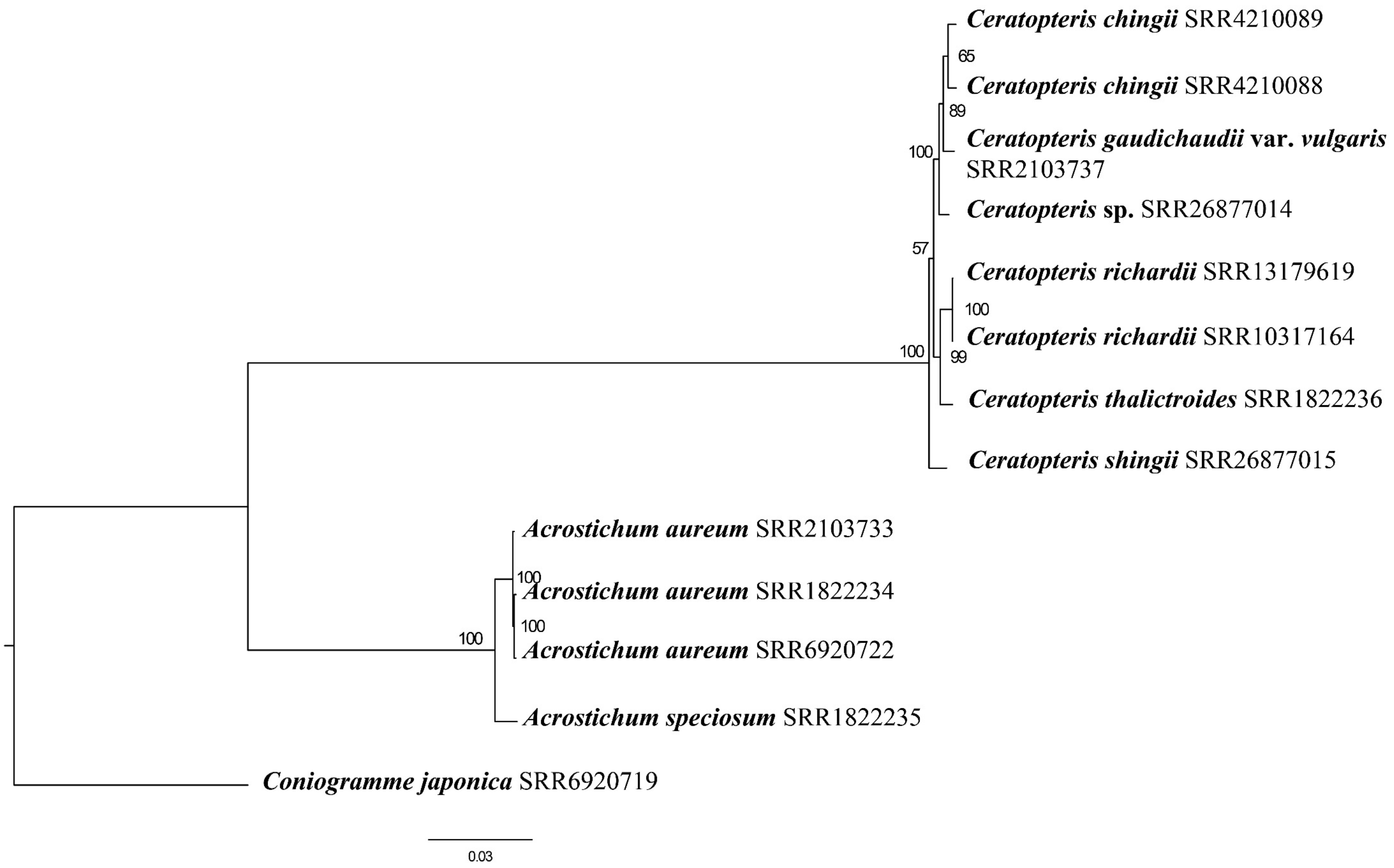

Figure S1. Phylogenetic tree constructed with 359 single-copy orthologous genes, which were merged into a supermatrix protein sequences with 127453 sites. The tree was constructed by RaxML under the best-fit model PROTGAMMAIJTTF found by ProtTest. Numbers on the branches are bootstrap values.

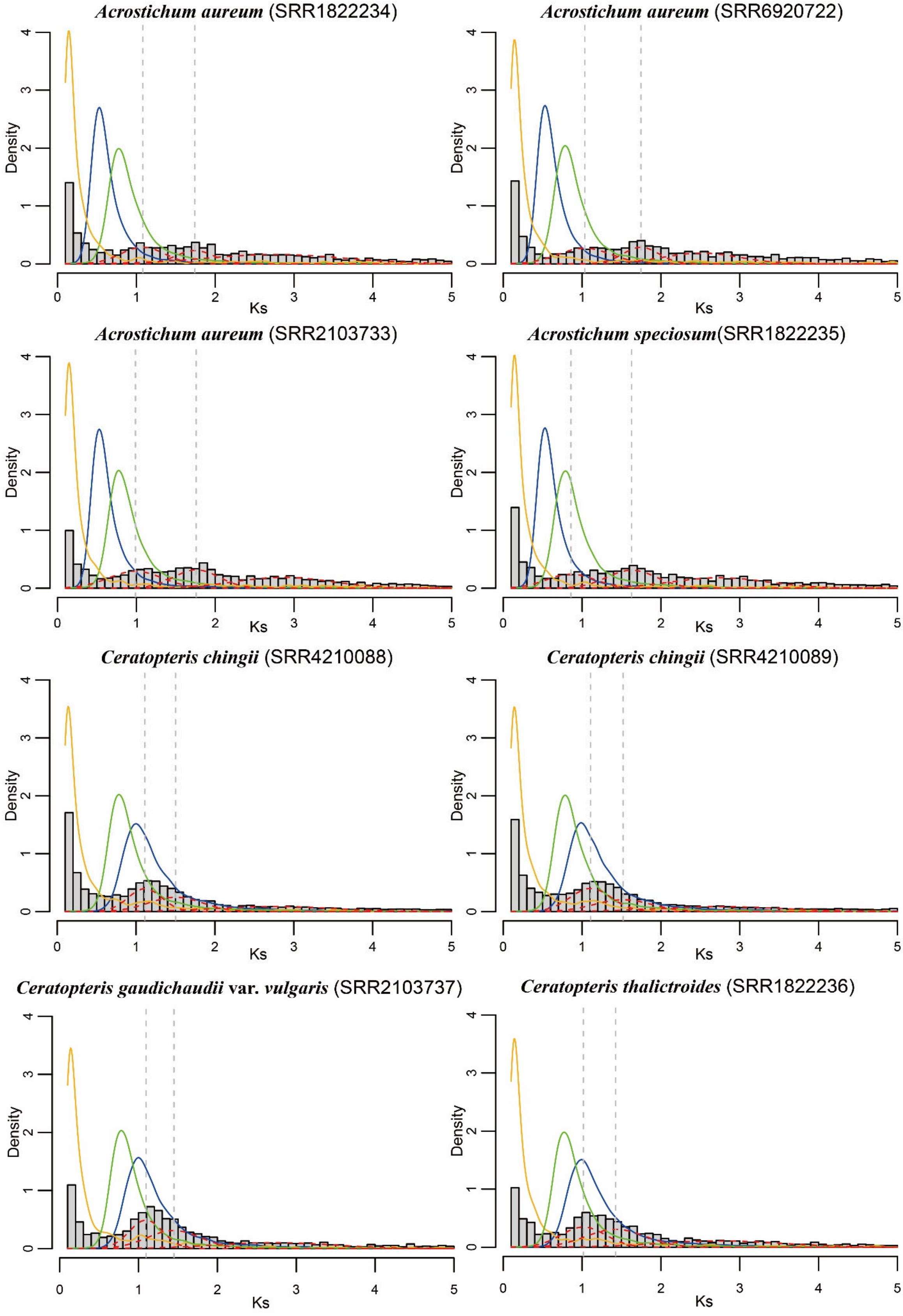

Acrostichum aureum (SRR1822234)
Acrostichum aureum (SRR6920722)
Acrostichum aureum (SRR2103733)
Acrostichum speciosum(SRR1822235)
Ceratopteris chingii (SRR4210088)
Ceratopteris chingii (SRR4210089)
Ceratopteris gaudichaudii var. *vulgaris* (SRR2103737)
Ceratopteris thalictroides (SRR1822236)
Density
Ks

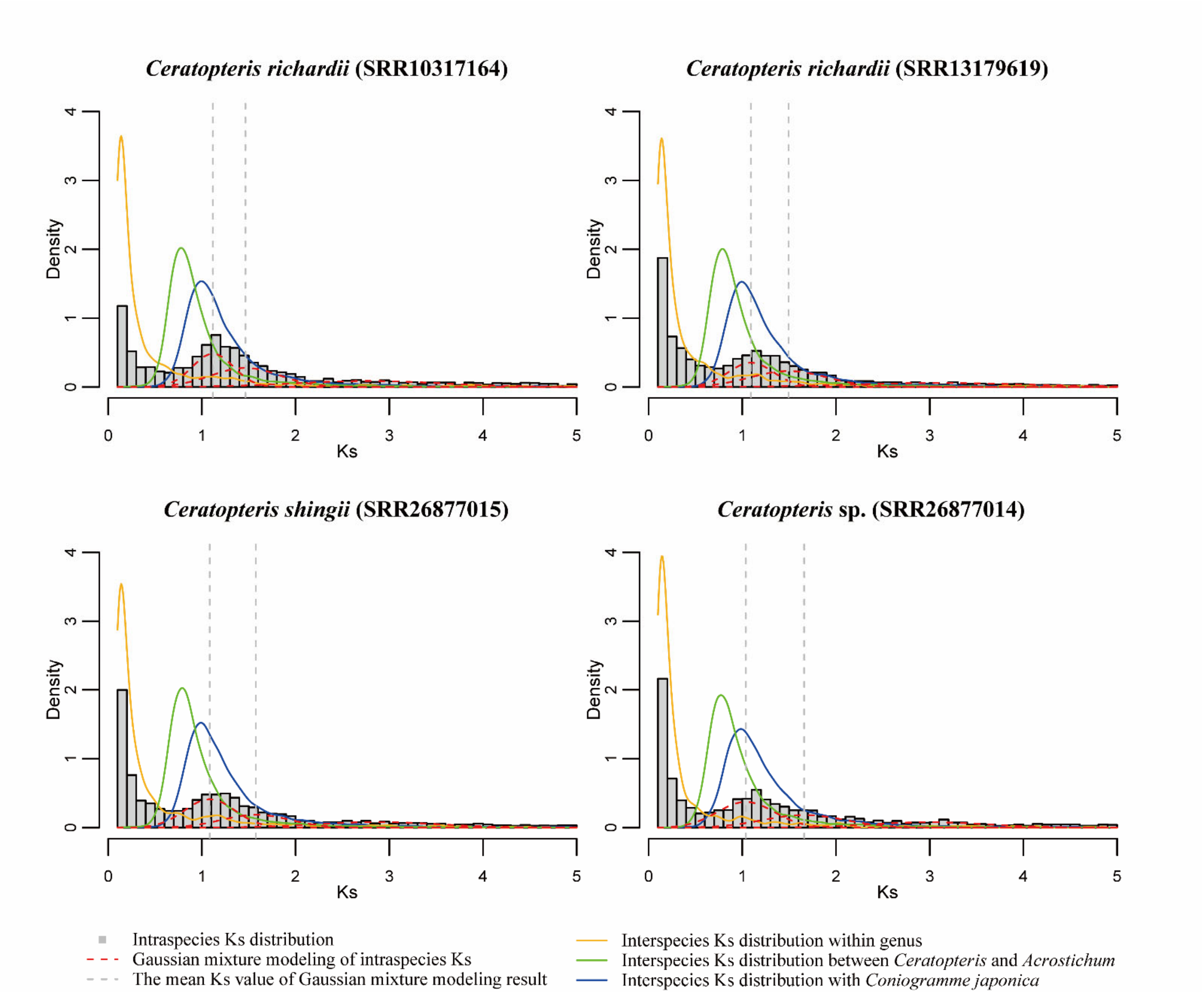

Figure S2. The plots of Ks analysis, including intraspecific and interspecific Ks analysis. The grey histograms presented the Ks distribution of intraspecific paralog pairs with a bin of 0.1 of x-axis, and the Gaussian mixture modeling results were curved in red dashed lines with the peaks marked by vertical grey dashed lines. The interspecific orthologues Ks distribution within genus (between species and *Cer*. *shingii* within *Ceratopteris*, between species and *Acr*. *speciosum* within *Acrostichum*), between genera (between the *Acrostichum* species and *Cer*. *shingii*, the *Ceratopteris* species and *Acr*. *speciosum*), with *Coniogramme japonica* (between species and *Con*. *japonica*) were presented by yellow, green, blue solid curves, respectively.

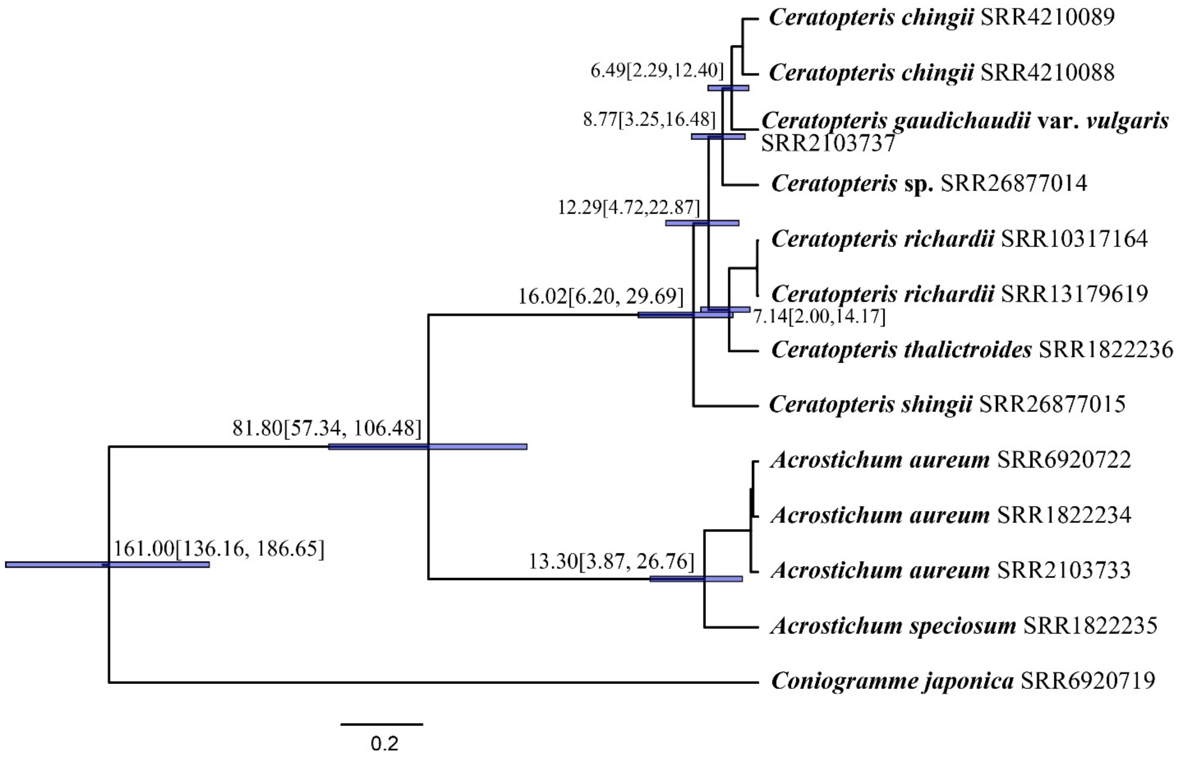

Figure S3. Divergence time estimation using 359 single-copy OGs. The numbers near each node are the corresponding estimated time with a confidence interval of 95%.

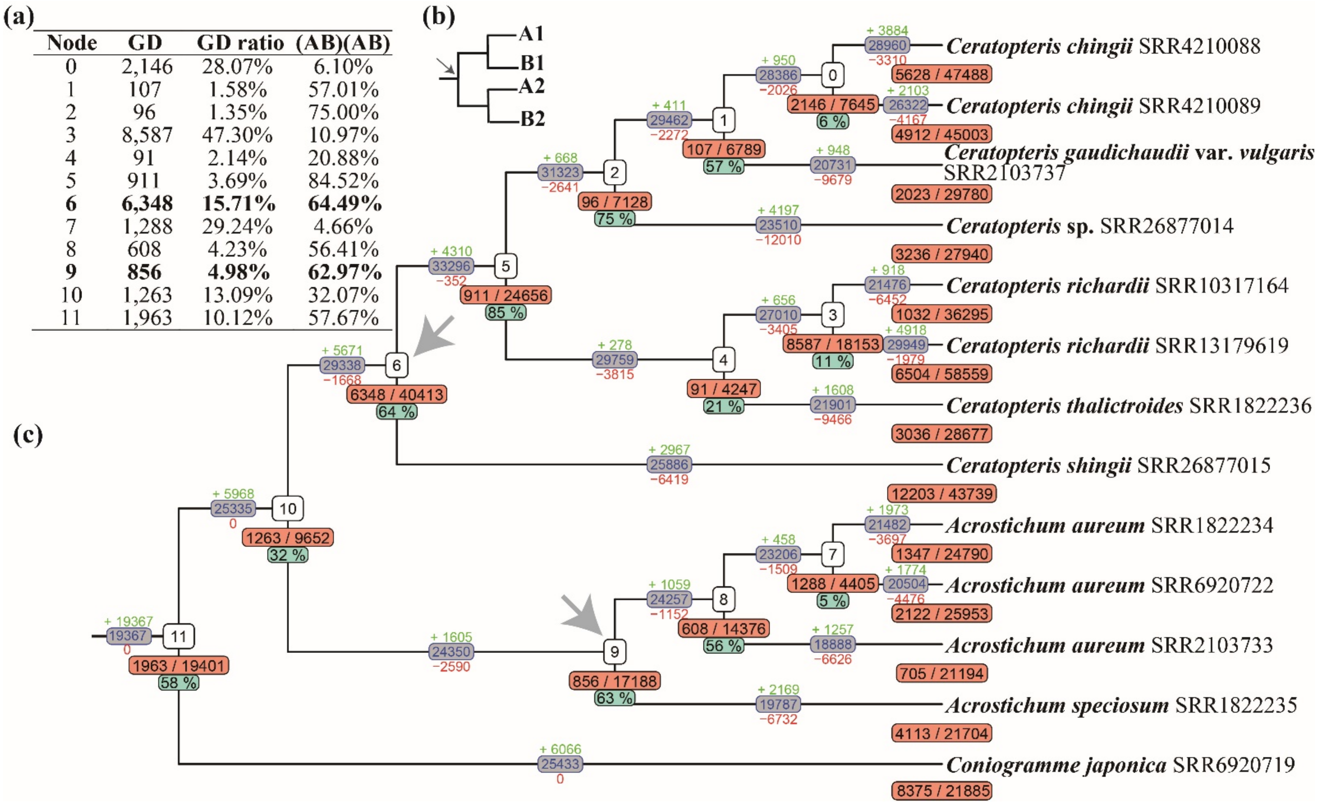

(a)

Node	GD	GD ratio	(AB)(AB)
0	2,146	28.07%	6.10%
1	107	1.58%	57.01%
2	96	1.35%	75.00%
3	8,587	47.30%	10.97%
4	91	2.14%	20.88%
5	911	3.69%	84.52%
**6**	**6,348**	**15.71%**	**64.49%**
7	1,288	29.24%	4.66%
8	608	4.23%	56.41%
**9**	**856**	**4.98%**	**62.97%**
10	1,263	13.09%	32.07%
11	1,963	10.12%	57.67%

Figure S4. Two WGD events inferred by the phylogenetic method. (a) The top-left table contains the number of duplicated gene families (GD), the ratio of GD to gene families (GD ratio), and the proportion of (AB)(AB) type GD of each node. The nodes that may experience WGD events are in bold. (b) A schematic tree of (AB)(AB) type GD. (c) In the tree file, the GD/gene families (yellow box) and the proportion of (AB)(AB) type GD (green box) are also marked below each node. The upper, along and below branch numbers, with green, blue and red colors, indicate the expansion and contraction of gene families. The putative WGD events are marked with grey arrows.

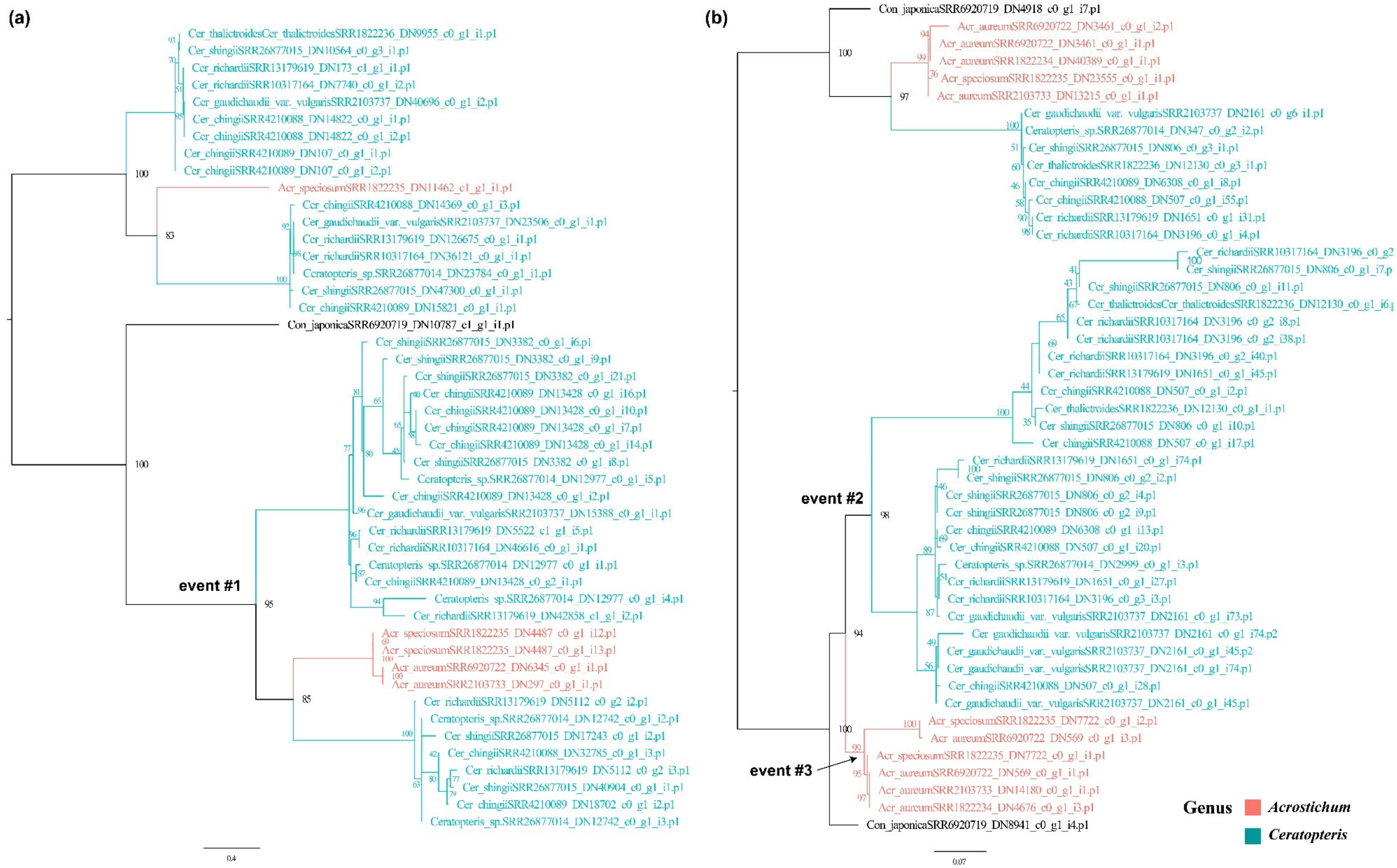

Figure S5. Phylogenetic gene trees of cluster 1109 (a) and cluster 1004 (b) constructed in Tree2GD. The two trees were selected randomly and could find the signals of three putative WGD events. The cluster 1109 and cluster 1004 contained 47 sequences of 12 samples, and 48 sequences of 13 samples, respectively. The gene trees were constructed by IQ-TREE with the JTT+G4 model and 1000 times ultrafast bootstrap. The detailed procedures can be find in the section of Materials and Methods. When inferred a duplicated gene (GD) candidate, the last common ancestor (LCA) of the two sub-branches should have depths with a difference of zero i.e. with topology as ((A,B), (A,B)) or a difference of one, i.e. with topology as ((A,B), B). The depth is calculated by the number of steps it takes to travel from the current node to the root on the species tree.

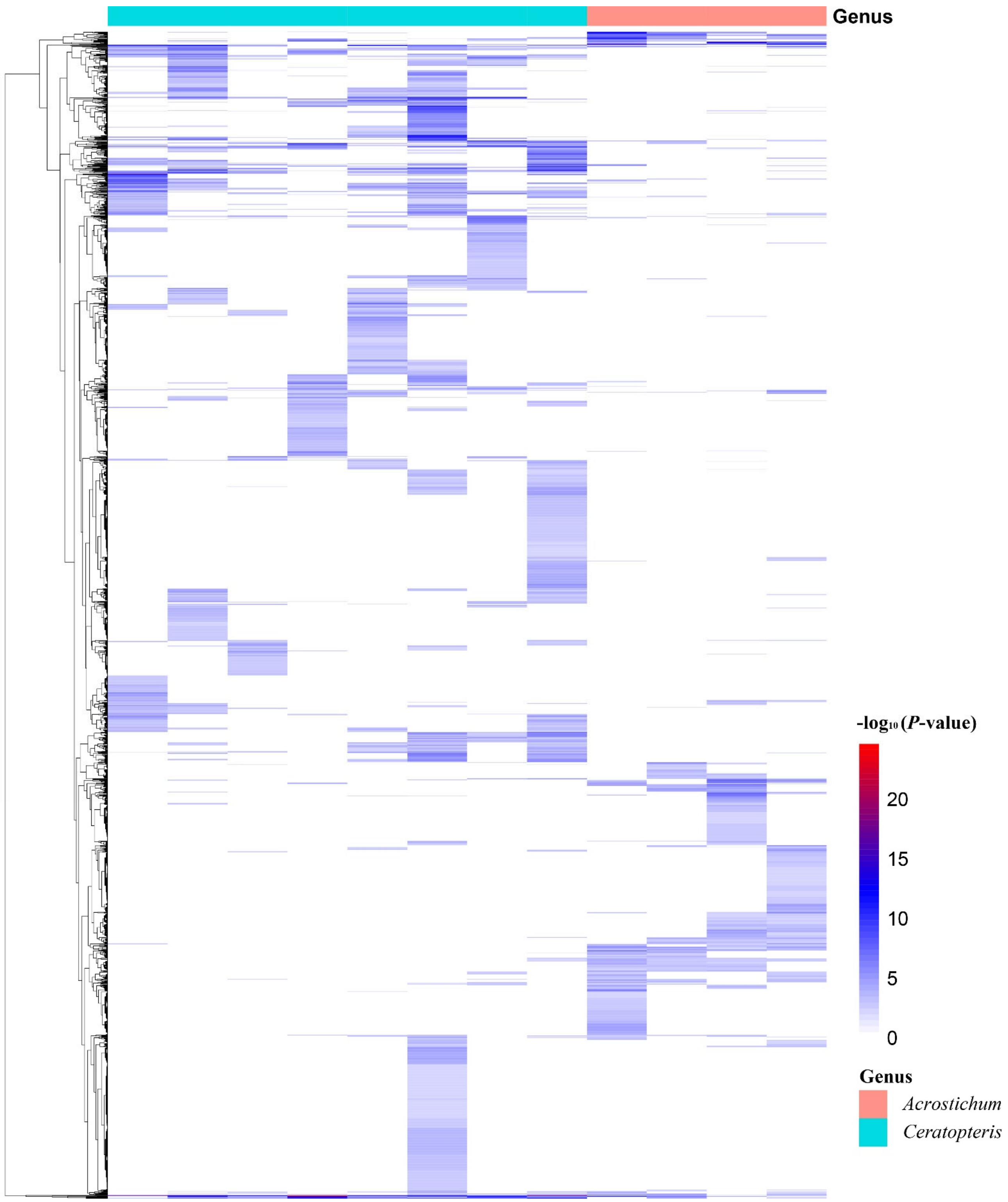

Figure S6. Functional enrichment heatmap of duplicated genes retained from two putative WGD events. The duplicated genes of each putative WGD event were functional enrichment analyzed, respectively, resulting in different patterns of functional terms.

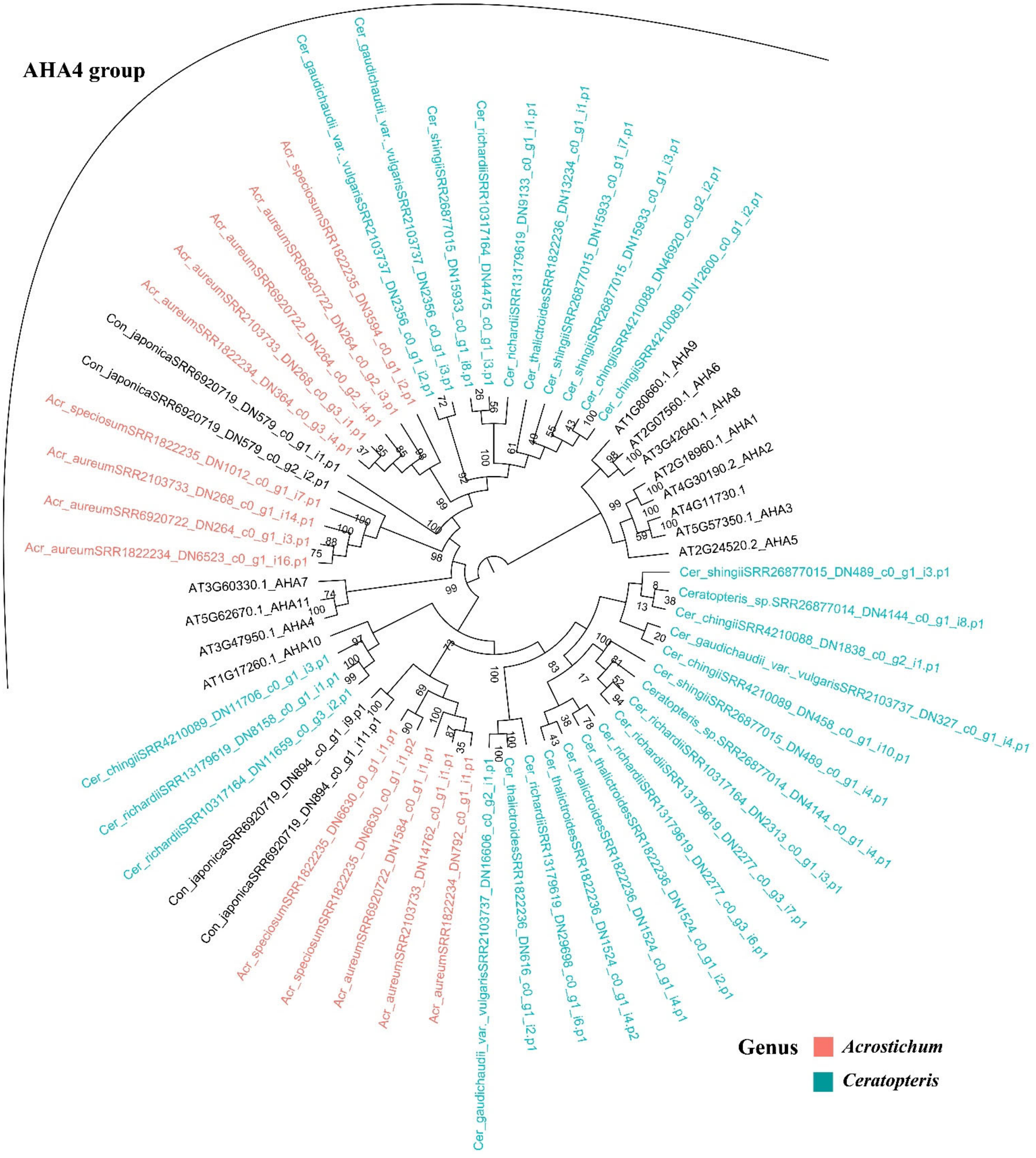

Figure S7. Phylogenetic tree of *AHA* genes. The alignment of *AHA* genes contained 59 protein sequences and 1077 sites. The tree was constructed by maximum likelihood method with the automatically determined substitution model (-m MFP) and 1000 bootstrapping repeats (-b 1000).

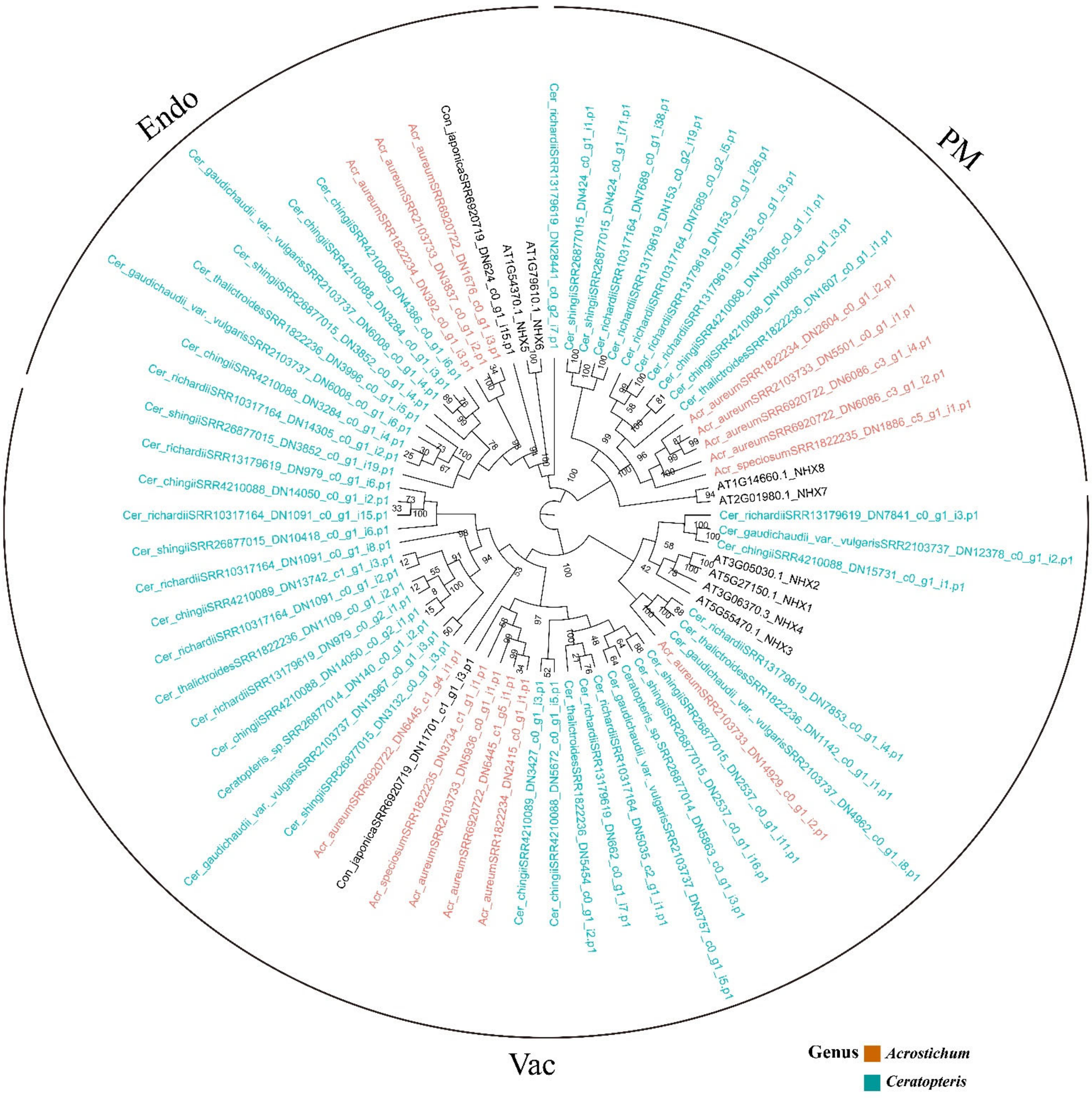

Figure S8. Phylogenetic tree of *NHX* genes. The alignment of *NHX* genes contained 72 protein sequences and 1313 sites. The tree was constructed by maximum likelihood method with the automatically determined substitution model (-m MFP) and 1000 bootstrapping repeats (-b 1000).

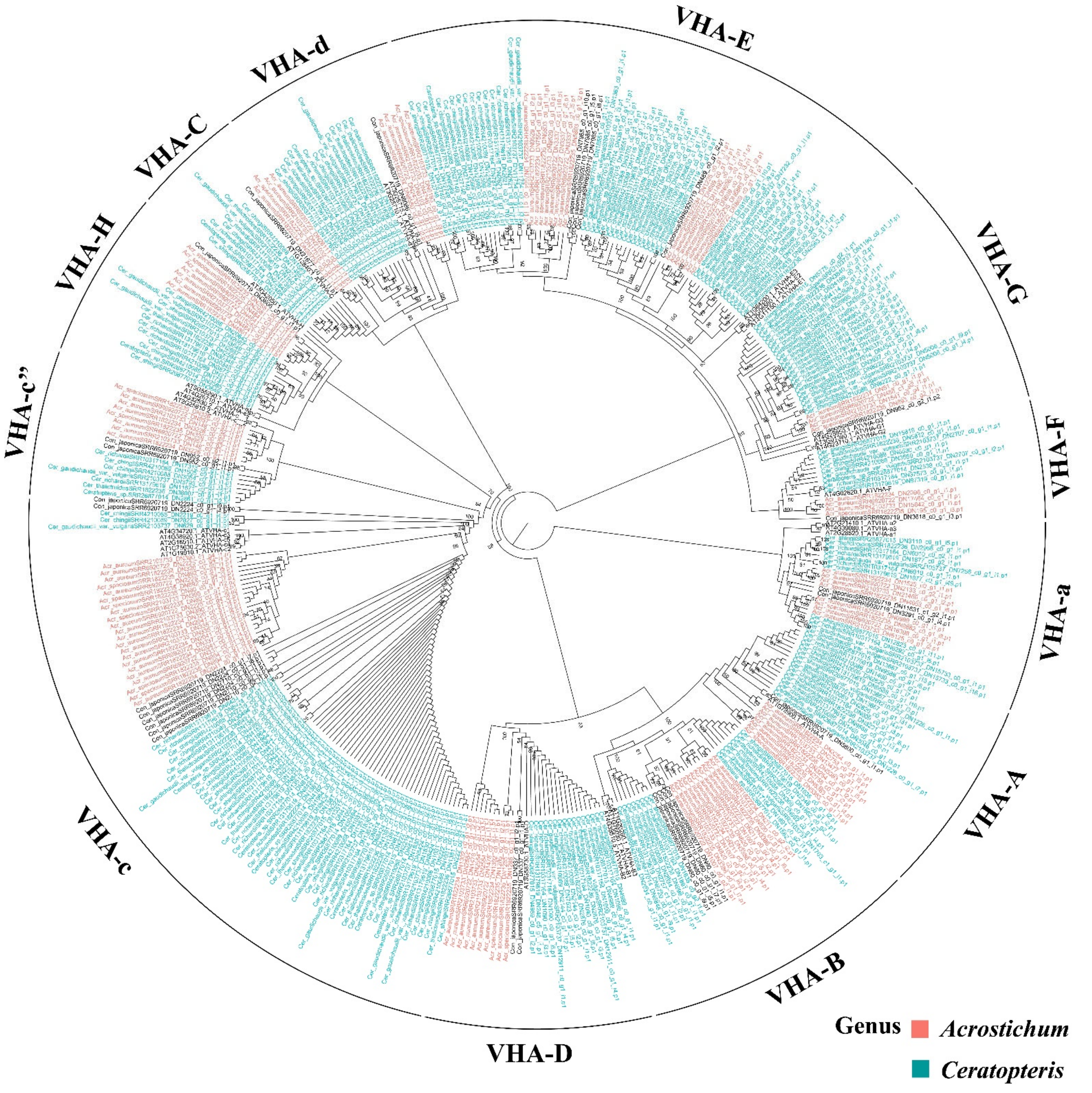

Figure S9. Phylogenetic tree of *VHA* genes. The alignment of *VHA* genes contained 399 protein sequences and 1239 sites. The tree was constructed by maximum likelihood method with the automatically determined substitution model (-m MFP) and 1000 bootstrapping repeats (-bb 1000).
